# Successful Management of Anomalous Lipton R-III Right Coronary Artery Chronic Total Occlusion

**DOI:** 10.7759/cureus.61505

**Published:** 2024-06-01

**Authors:** Ankit Gupta, Sreenivas Reddy, Ashish Jain, Bhushan Shah

**Affiliations:** 1 Department of Cardiology, All India Institute of Medical Sciences, Raebareli, Raebareli, IND; 2 Department of Cardiology, Government Medical College and Hospital, Chandigarh, IND; 3 Department of Cardiology, All India Institute of Medical Sciences, Bhopal, Bhopal, IND

**Keywords:** anomalous coronary artery, single sinus, single ostium, angiography, chronic total occlusion, percutaneous coronary intervention, coronary artery anomaly

## Abstract

Chronic total occlusion (CTO) of the coronary artery is a subset where cardiologists confront technical challenges most of the time during percutaneous coronary intervention (PCI). A congenital coronary anomaly is considered a critical challenge, especially when accompanied by CTO lesions. We report a case of a 64-year-old hypertensive and chronic smoker male who presented to our tertiary care center with chief complaints of Canadian Cardiovascular Society II angina. Coronary angiography revealed proximal right coronary artery CTO in a patient with an anomalous origin of coronary arteries arising from the right single sinus “Lipton R-III” which was managed successfully through PCI.

## Introduction

Among congenital coronary artery anomalies, both the arteries arising from a single coronary ostium is a very rare angiographic finding, occurring in around 0.02-0.06% of the general population [1]. Many of these anomalies are clinically benign; however, others are associated with serious morbidity [2]. Coronary intervention for an anomalous coronary artery is technically challenging, especially in cases with chronic total occlusion (CTO). In addition, technical complexities are associated with CTO at every step, from engaging the coronary ostium to delivery of hardware through the vessel [3]. Selecting an appropriate guide catheter is crucial to ensure selective angiography, assessment of obstructive lesions, and adequate support during percutaneous coronary intervention (PCI) [4]. This report presents a novel proximal right coronary artery (RCA) CTO case in a patient with an anomalous origin of coronary arteries arising from a single sinus managed successfully through PCI. This article was previously presented as a meeting abstract at TCTAP 24 Korea on April 26, 2024.

## Case presentation

**Figure 1:**
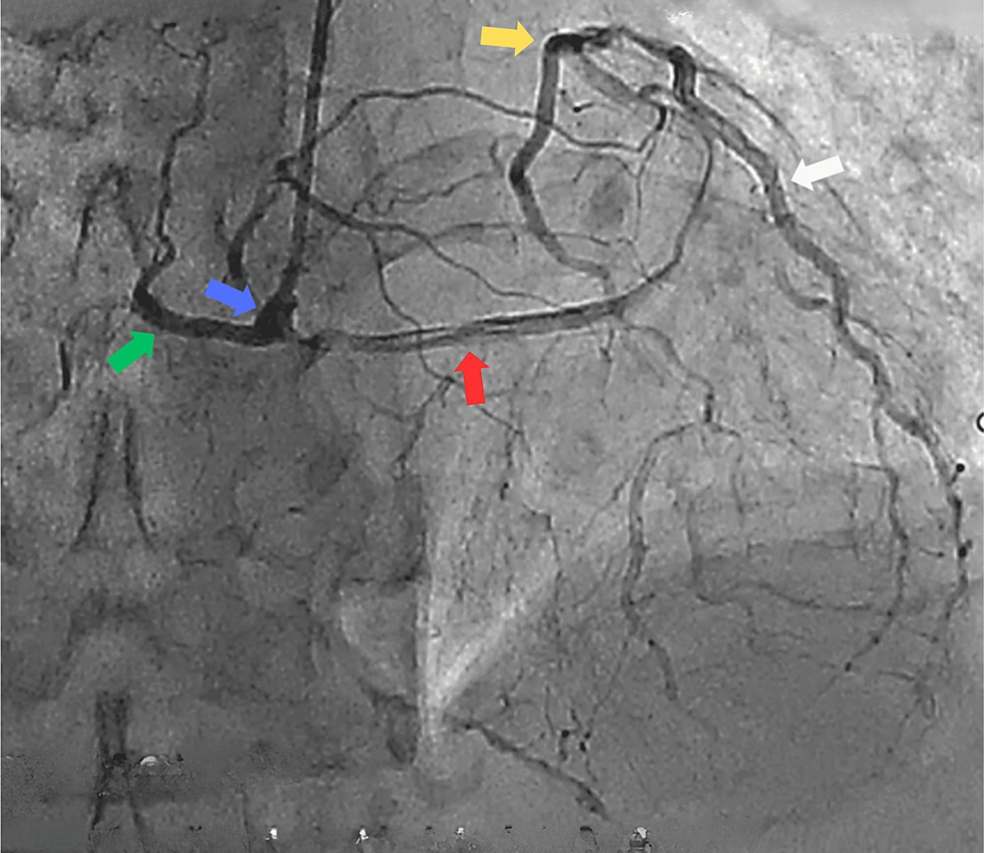
Angiogram showing anomalous left (red arrow) and right (green arrow) coronary arteries arising from a single sinus (blue arrow), normal left main, diffused plaque in the LAD artery (yellow arrow), plaque at the proximal LCX artery (white arrow), ostial obtuse marginal branch, dominant RCA with existence of CTO at proximal RCA and retrogradely filling from the left coronary arteries RCA: right coronary artery, CTO: chronic total occlusion, LAD: left anterior descending, LCX: left circumflex

**Figure 2:**
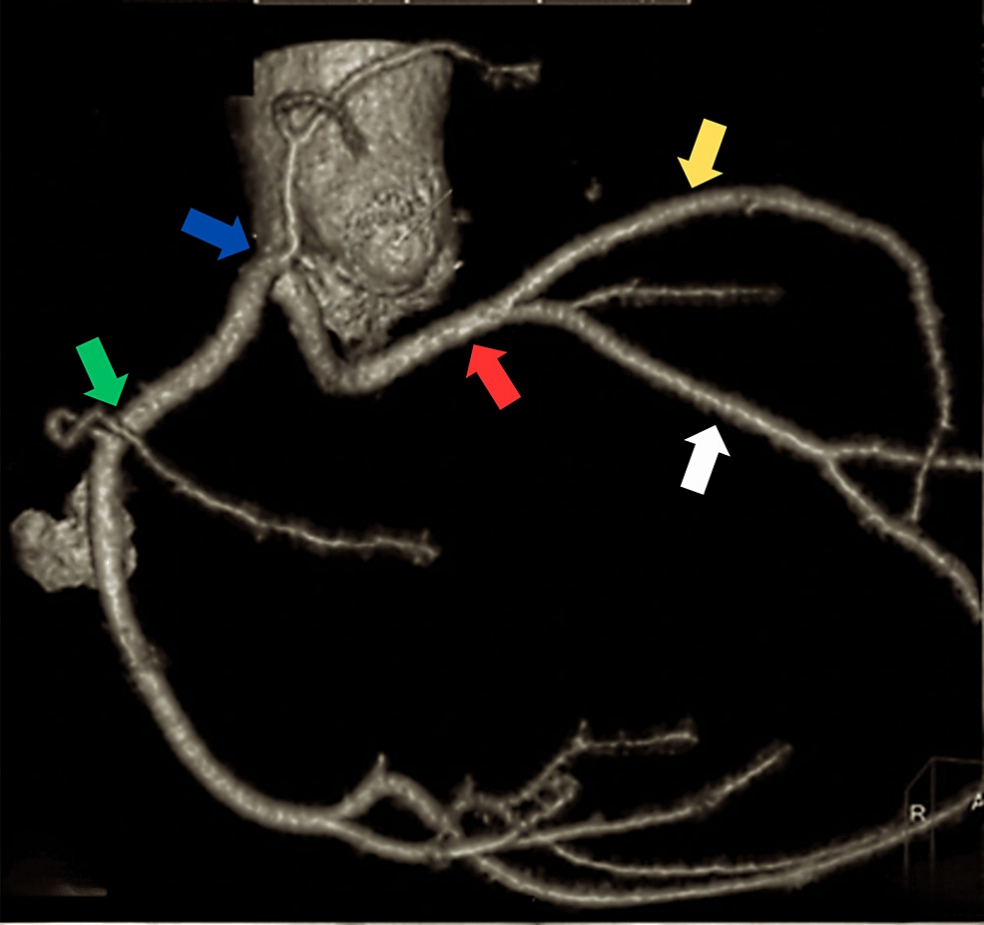
A computed tomography angiogram showing anomalous left (red arrow) and right (green arrow) coronary arteries arising from a single sinus (blue arrow), LAD artery (yellow arrow), and LCX artery (white arrow) LAD: left anterior descending, LCX: left circumflex

A 64-year-old hypertensive and chronic smoker male presented to our tertiary care center with chief complaints of Canadian Cardiovascular Society II angina. The patient had a medical history of PCI to RCA CTO one year ago and has had breathlessness for the past two weeks. An echocardiogram revealed a 55% left ventricular ejection fraction, left ventricular diastolic dysfunction, and left ventricular hypertrophy. An angiogram revealed anomalous left and right coronary arteries arising from a single sinus, normal long left main running straight in the right anterior oblique view, suggesting running between the pulmonary artery and aorta, with a diffuse plaque in the left anterior descending artery, plaque in the left circumflex artery, and ostial plaque in the first obtuse marginal branch. The patient was diagnosed with a single-vessel disease. The RCA was dominant, representing the CTO of the proximal segment, which was retrogradely filling from the left system (Figure 1). His computed tomography coronary angiogram revealed the course of the long left main, originating from the right single sinus running between the aorta and pulmonary artery (Figure 2).

**Figure 3:**
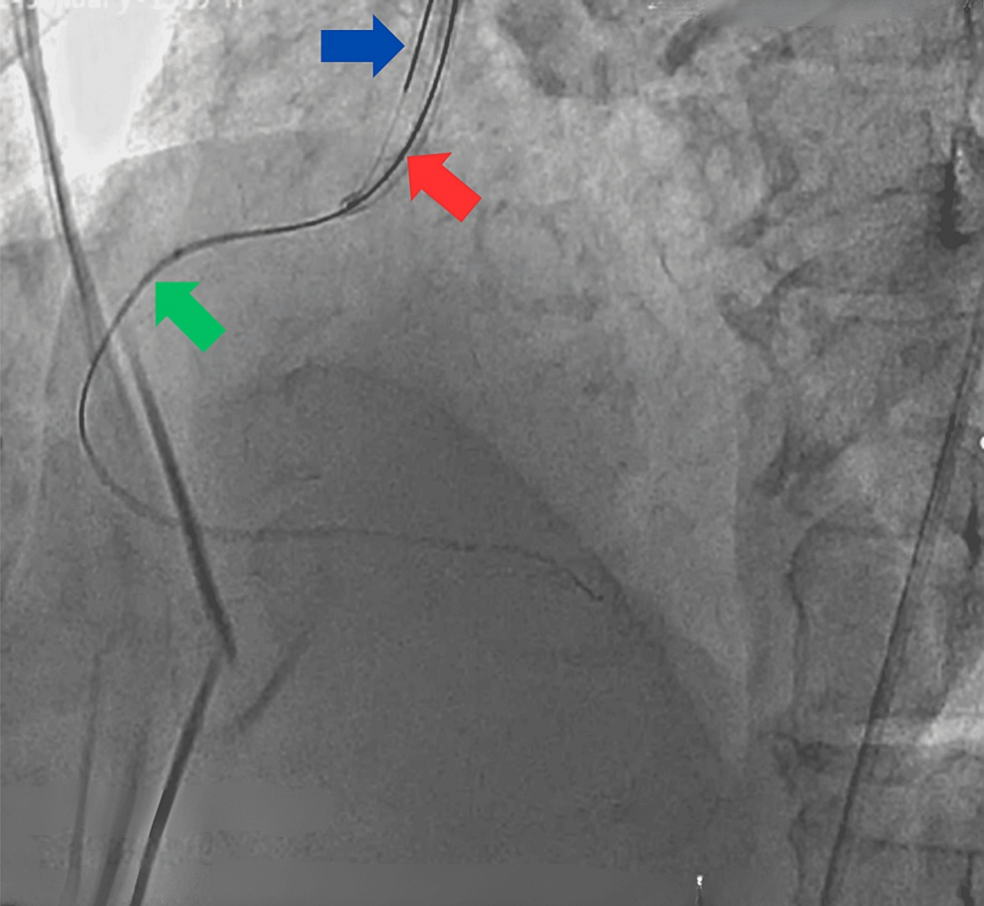
Angioplasty showing the Fielder XT wire (red arrow) used to cross the lesion and another wire (blue arrow) placed in the aorta. The green arrow shows the RCA RCA: right coronary artery

As the long left main was normal with insignificant lesions, the heart team made a cumulative decision to attempt RCA CTO with an antegrade approach. The right sinus was engaged with the JR 3.5/6F guiding catheter via the right femoral approach, with the support of a floppy wire. Firstly, the micro-catheter end hole was kept at the proximal cap of the CTO lesion at the proximal segment of the RCA. After that, using multiple hard-tip wires, we crossed the lesion with a Fielder XT wire (Asahi Intecc Co., Ltd., Japan) using the parallel wire technique. We kept the Fielder XT in the posterior descending artery, but the micro-catheter failed to progress beyond the proximal segment due to fibrocalcification. Hence, another floppy wire was placed in the aorta to secure the safety of the single sinus (Figure 3).

**Figure 4:**
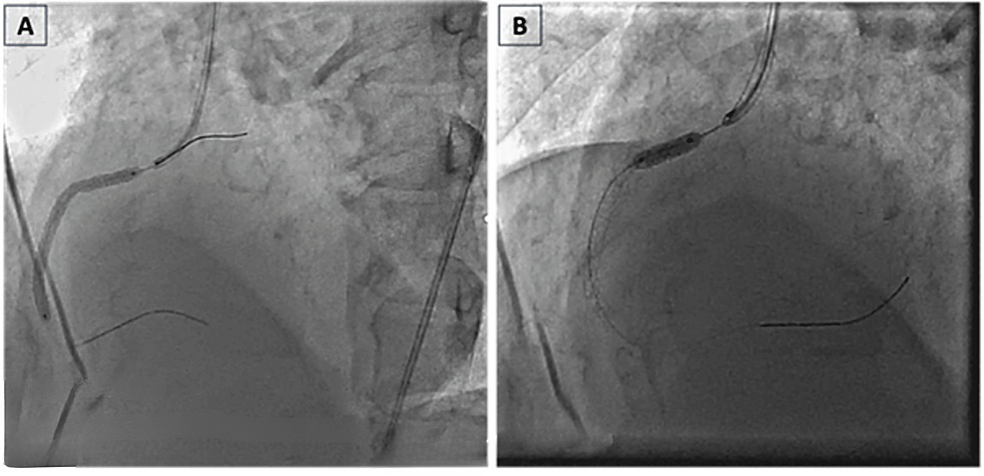
Angioplasty showing (A) a 3.0 × 48 mm VIVO ISAR drug-eluting stent negotiated and deployed in the proximal to mid-RCA at 12 atm and (B) post-dilatation in the proximal to mid-RCA using a 3.25 × 12 mm Pipit NC balloon at 12–14 atm RCA: right coronary artery

**Figure 5:**
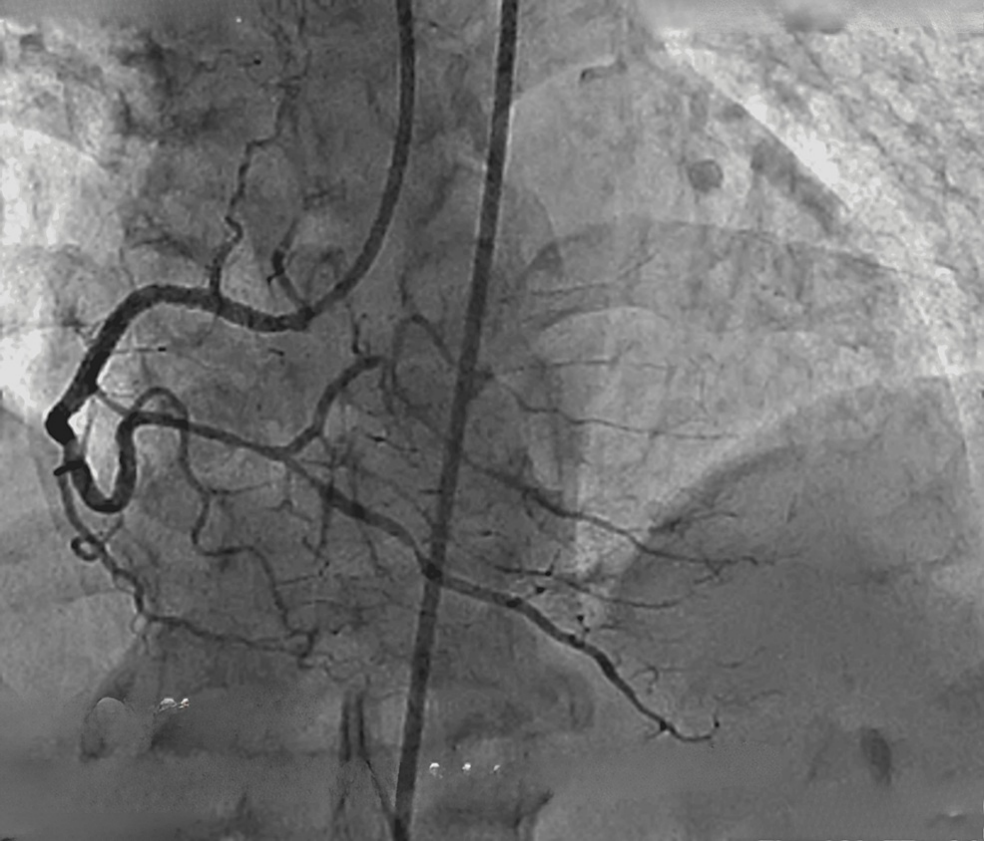
Post-stent thrombolysis in myocardial infarction flow

As the micro-catheter failed to progress, we took an Alveo HP CTO balloon (BrosMed Medical Co., Ltd, Guangdong, China) and gently moved toward the lesion. Balloon dilatation was done using a 0.75 × 6 mm balloon at 20 atm to break the fibro-calcific proximal cap to create a breezy channel. After various efforts, the CTO balloon crossed the lesion, and sequential pre-dilatation was performed. After that, the lesion was sequentially predilated using a 1.5 × 15 mm Mini Trek balloon (Abbott Vascular, Santa Clara, CA, USA) and a 2.5 × 15 mm Trek balloon (Abbott Vascular, Santa Clara, CA, USA) at 12-16 atm after exchanging the Fielder XT with SION blue workhorse wire (Asahi Intecc Co., Ltd., Japan). A 3.0 × 48 mm VIVO ISAR drug-eluting stent (Translumina Therapeutics LLP, Dehradun, India) was positioned and deployed in the proximal to initial distal RCA at 20 atm high pressure (Figure 4a) and was subsequently post-dilatated using a 3.25 × 12 mm Pipit noncompliant balloon (Sahajanand Medical Technologies Ltd., Surat, India) with high pressure at 20-28 atm (Figure 4b). The final check angiogram revealed TIMI-3 flow (Figure 5). The procedure went uneventfully. The patient was hemodynamically stable and was discharged after 48 hours.

## Discussion

The prevalence of anomalous origin both of left and right coronary arteries from a single coronary ostium is relatively rare, and the exact prevalence can vary among different populations and studies. However, it is generally estimated to occur in <1% of the general population.

It is important to note that coronary artery anomalies, including alterations in the number and origin of coronary ostia, can manifest in various forms. Some individuals may have no symptoms related to these anomalies. In contrast, others may experience cardiac symptoms or complications such as sudden death and ischemia, which could be the result of compression by the aorta and pulmonary artery [1,5]. The coronary circulation arising from a single coronary ostium has little clinical significance, except for cases in which a coronary artery traverses between the pulmonary artery and aorta, which can cause sudden death at a young age due to extrinsic coronary arterial occlusion [6].

CTO lesions present a challenging aspect of coronary intervention. The choice between an antegrade or retrograde approach during CTO PCI depends on the specific anatomy of the coronary arteries, the location and length of the CTO, the availability of collateral vessels for retrograde access, and the expertise of the interventional cardiologist. Yamada et al. documented a similar case of retrograde coronary intervention for a CTO at the ostium of the RCA with an anomalous origin, highlighting the potential for retrograde techniques to effectively treat CTOs even in cases of coronary artery anomalies [2]. Similarly, Cocco et al. presented a case of PCI to CTO lesion in an anomalous RCA. During the procedure, an unexpected rupture of the coronary artery occurred after dilatation with a small balloon at low pressure, leading to unforeseen insights. Despite this complication, successful CTO treatment was achieved [7]. However, Cocco et al. reported a warning on the risk of complications during complex PCI of anomalous arteries. They hypothesized that the artery wall could be fragile due to histopathological alterations, which could have a role in the pathophysiology of coronary malignancy [7]. In the present case, despite a CTO lesion in the proximal RCA, blood flow to the distal portion of the RCA was maintained through retrograde flow from the left coronary system. This retrograde flow may occur through collateral vessels or alternative pathways.

Managing a proximal CTO is a challenging procedure on its own, but the presence of an anomalous coronary artery adds an extra layer of complexity to it. The operator had to adapt their approach to address both issues simultaneously. The combination of CTO with anomalous left and right coronary arteries arising from a single ostium in this patient highlights the expertise of the medical team and their ability to adapt established techniques to address complex and distinctive clinical situations. This case could contribute to the medical literature in terms of its approach and successful outcome in managing such complex cardiovascular issues in a single procedure. Post-discharge, the patient was asymptomatic at the 22-day follow-up.

## Conclusions

This case highlights the challenges encountered during PCI, such as the difficulty in advancing the microcatheter due to fibrocalcification. A floppy wire is placed in the aorta to secure the safety of the single sinus, which is a crucial safety measure.

The successful treatment of this critical case may have important clinical implications for the patient's cardiac health and overall well-being, making it a valuable learning experience for the medical community. The novelty of this case arises from its rare and complex combination of anatomical variations, clinical challenges, and the need for advanced interventional techniques.
